# The banana genome hub: a community database for genomics in the Musaceae

**DOI:** 10.1093/hr/uhac221

**Published:** 2022-09-28

**Authors:** Gaëtan Droc, Guillaume Martin, Valentin Guignon, Marilyne Summo, Guilhem Sempéré, Eloi Durant, Alexandre Soriano, Franc-Christophe Baurens, Alberto Cenci, Catherine Breton, Trushar Shah, Jean-Marc Aury, Xue-Jun Ge, Pat Heslop Harrison, Nabila Yahiaoui, Angélique D’Hont, Mathieu Rouard

**Affiliations:** CIRAD, UMR AGAP Institut, F-34398 Montpellier, France; UMR AGAP Institut, Univ Montpellier, CIRAD, INRAE, Institut Agro, F-34398 Montpellier, France; French Institute of Bioinformatics (IFB) - South Green Bioinformatics Platform, Bioversity, CIRAD, INRAE, IRD, F-34398 Montpellier, France; CIRAD, UMR AGAP Institut, F-34398 Montpellier, France; UMR AGAP Institut, Univ Montpellier, CIRAD, INRAE, Institut Agro, F-34398 Montpellier, France; French Institute of Bioinformatics (IFB) - South Green Bioinformatics Platform, Bioversity, CIRAD, INRAE, IRD, F-34398 Montpellier, France; French Institute of Bioinformatics (IFB) - South Green Bioinformatics Platform, Bioversity, CIRAD, INRAE, IRD, F-34398 Montpellier, France; Bioversity International, Parc Scientifique Agropolis II, 34397 Montpellier, France; CIRAD, UMR AGAP Institut, F-34398 Montpellier, France; UMR AGAP Institut, Univ Montpellier, CIRAD, INRAE, Institut Agro, F-34398 Montpellier, France; French Institute of Bioinformatics (IFB) - South Green Bioinformatics Platform, Bioversity, CIRAD, INRAE, IRD, F-34398 Montpellier, France; French Institute of Bioinformatics (IFB) - South Green Bioinformatics Platform, Bioversity, CIRAD, INRAE, IRD, F-34398 Montpellier, France; CIRAD, UMR INTERTRYP, F-34398 Montpellier, France; INTERTRYP, Université de Montpellier, CIRAD, IRD, 34398 Montpellier, France; French Institute of Bioinformatics (IFB) - South Green Bioinformatics Platform, Bioversity, CIRAD, INRAE, IRD, F-34398 Montpellier, France; Syngenta Seeds SAS, Saint-Sauveur, 31790, France; DIADE, Univ Montpellier, CIRAD, IRD, Montpellier, 34830, France; CIRAD, UMR AGAP Institut, F-34398 Montpellier, France; UMR AGAP Institut, Univ Montpellier, CIRAD, INRAE, Institut Agro, F-34398 Montpellier, France; French Institute of Bioinformatics (IFB) - South Green Bioinformatics Platform, Bioversity, CIRAD, INRAE, IRD, F-34398 Montpellier, France; CIRAD, UMR AGAP Institut, F-34398 Montpellier, France; UMR AGAP Institut, Univ Montpellier, CIRAD, INRAE, Institut Agro, F-34398 Montpellier, France; French Institute of Bioinformatics (IFB) - South Green Bioinformatics Platform, Bioversity, CIRAD, INRAE, IRD, F-34398 Montpellier, France; Bioversity International, Parc Scientifique Agropolis II, 34397 Montpellier, France; French Institute of Bioinformatics (IFB) - South Green Bioinformatics Platform, Bioversity, CIRAD, INRAE, IRD, F-34398 Montpellier, France; Bioversity International, Parc Scientifique Agropolis II, 34397 Montpellier, France; IITA, Nairobi P.O. Box 30709-00100, Kenya; Génomique Métabolique, Genoscope, Institut François Jacob, CEA, CNRS, Univ Evry, Université Paris-Saclay, 2 rue Gaston Crémieux, 91057 Evry, France; Key Laboratory of Plant Resources Conservation and Sustainable Utilization, South China Botanical Garden, Chinese Academy of Sciences, Guangzhou 510520, China; Center of Conservation Biology, Core Botanical Gardens, Chinese Academy of Sciences, Guangzhou 510520, China; Key Laboratory of Plant Resources Conservation and Sustainable Utilization, South China Botanical Garden, Chinese Academy of Sciences, Guangzhou 510520, China; Department of Genetics and Genome Biology, University of Leicester, Leicester LE1 7RH, UK; CIRAD, UMR AGAP Institut, F-34398 Montpellier, France; UMR AGAP Institut, Univ Montpellier, CIRAD, INRAE, Institut Agro, F-34398 Montpellier, France; CIRAD, UMR AGAP Institut, F-34398 Montpellier, France; UMR AGAP Institut, Univ Montpellier, CIRAD, INRAE, Institut Agro, F-34398 Montpellier, France; French Institute of Bioinformatics (IFB) - South Green Bioinformatics Platform, Bioversity, CIRAD, INRAE, IRD, F-34398 Montpellier, France; Bioversity International, Parc Scientifique Agropolis II, 34397 Montpellier, France

## Abstract

The Banana Genome Hub provides centralized access for genome assemblies, annotations, and the extensive related omics resources available for bananas and banana relatives. A series of tools and unique interfaces are implemented to harness the potential of genomics in bananas, leveraging the power of comparative analysis, while recognizing the differences between datasets. Besides effective genomic tools like BLAST and the JBrowse genome browser, additional interfaces enable advanced gene search and gene family analyses including multiple alignments and phylogenies. A synteny viewer enables the comparison of genome structures between chromosome-scale assemblies. Interfaces for differential expression analyses, metabolic pathways and GO enrichment were also added. A catalogue of variants spanning the banana diversity is made available for exploration, filtering, and export to a wide variety of software. Furthermore, we implemented new ways to graphically explore gene presence-absence in pangenomes as well as genome ancestry mosaics for cultivated bananas. Besides, to guide the community in future sequencing efforts, we provide recommendations for nomenclature of locus tags and a curated list of public genomic resources (assemblies, resequencing, high density genotyping) and upcoming resources—planned, ongoing or not yet public. The Banana Genome Hub aims at supporting the banana scientific community for basic, translational, and applied research and can be accessed at https://banana-genome-hub.southgreen.fr.

## Introduction

The *Musaceae,* known as the banana family, belongs to the monocotyledons, that comprise crops of great economic value as well as ornamental plants. Notably, *Musaceae* includes the genus *Musa* with bananas, a top-ten crop for food security, and arguably the favorite fruit worldwide [1]. Its sister genus, Ensete, contains *Ensete ventricosum,* an important crop for food security in Ethiopia [2] and ornamental plants like *Ensete glaucum* widely distributed in Asia. The final monospecific genus in *Musaceae* includes *Musella lasiocarpa* from southwest China and possibly extinct in the wild*.* Wild species within *Musaceae* are diploids, with basic chromosome numbers of x = 9, 10 and 11. The *Musa* cultivars grown for fruit result from hybridization between different wild diploid *Musa* species and subspecies. They are parthenocarpic, sterile or poorly fertile and mostly cultivated as vegetatively propagated triploids (2n = 3x = 33) although some cultivars are diploids or tetraploids, most of cultivars bear large structural variations in their chromosomes, transmitted from different wild ancestors. All these features make banana breeding very complex. Genomic characterization has a great potential to significantly contribute to better conservation strategies, improved use of banana genetic resources and increased sustainability of crop production [3, 4]. Increasing the availability of genomic resources and facilitating their use has been much needed [5, 6].

In 2012, the first Musaceae reference genome, representative of *Musa acuminata* (A genome), was published [7] alongside the Banana Genome Hub [8] (https://banana-genome-hub.southgreen.fr). In the last decade, this reference was iteratively improved [9, 10] while a number of new genome assemblies of different *Musaceae* species have also been generated. The next sequenced genome was that of *Musa balbisiana* (B genome) [11], first as a draft genome and later as a chromosome-scale assembly from a double haploid [12]. In the meantime, draft assemblies of *Musa itinerans* [13], *E. ventricosum* [14], *Musa textilis* [15] and other subspecies of *M. acuminata* were produced [16]. A pangenome composed of the 15 individuals belonging to Ensete and Musa was also developed [17]. Benefiting from easier and cheaper access to long reads sequencing technologies and scaffolding methods, chromosome scale genome assemblies were released for *Musa schizocarpa* [18], *Ensete glaucum* [19] and a telomere-to-telomere assembly of *M. acuminata* was published [10]. Thanks to available reference genomes, a broad range of studies have been conducted to explore multiple aspects including genetic diversity [20], plant genome evolution [21–23], chromosome structural variation [24], gene family analyses [25–28], trait-phenotype [29, 30], association genetics [31–33] and genetic engineering [34]. All these topics need access to various types of datasets and related query or visualisation interfaces.

Here, we present an overhauled and enriched version of the Banana Genome Hub (BGH), a community database that serves as a central online platform for whole genome sequences and related omics data on *Musaceae.* We detail the implemented interfaces, and the way data were collected and curated. Finally, we list and discuss the status of sequencing projects and propose a locus name nomenclature for future projects about the genomics of *Musaceae*.

## Tools and interfaces

**Figure 1:**
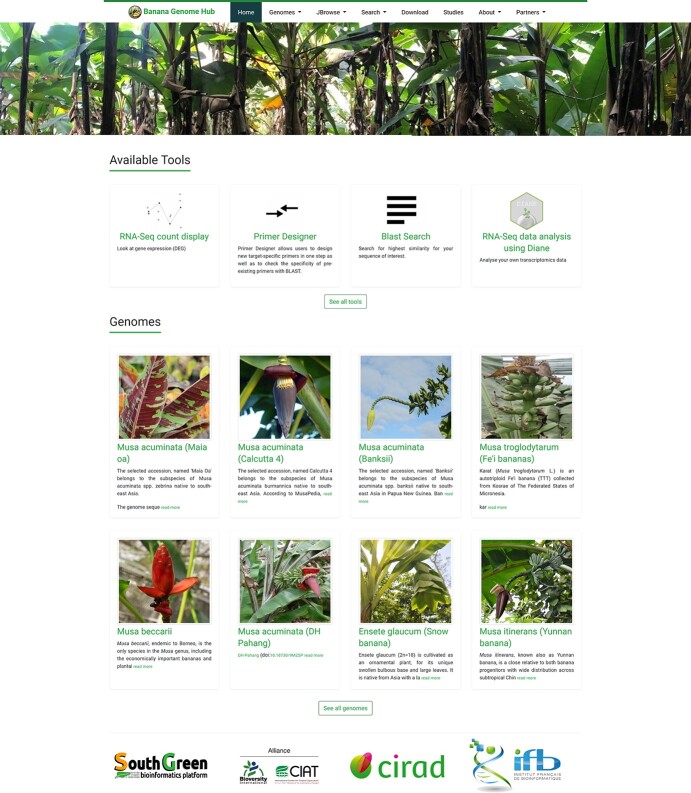
Screenshot of the Banana Genome Hub homepage showing a subset of available genome sequence and visualisation and analytical tools.

We implemented a list of web interface and collected data to facilitate functional and comparative genomics-oriented data analyses (Figure 1). Some interfaces focus on exploration of individual genes or of a list of genes to check their location on the genome, presence in gene families, their expression patterns, their functional annotations (i.e. Gene Ontologoy (GO)) as well as associated SNP markers. Other tools enable a more global exploration of chromosome structures by looking at synteny, presence absence variation and genome ancestry mosaics. From a technical perspective, the BGH core has been developed with the Tripal toolkit (i.e. Drupal v7, Tripal v3), an open-source project supporting the development of biological databases [8, 35, 36] complemented by the development of additional modules [37]. All these elements are further described below.

### Gene(s) query including orthogroups and omics-related datasets

**Figure 2:**
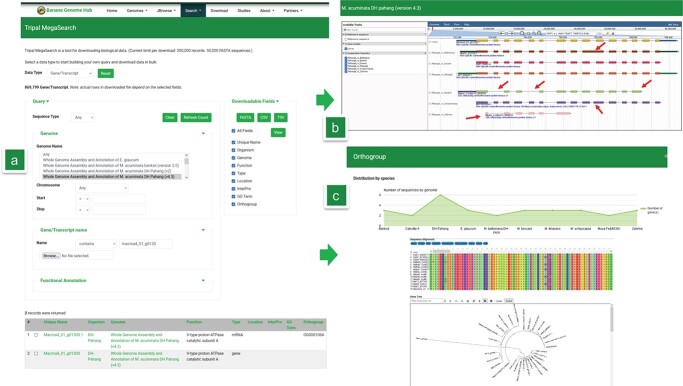
(A) Gene search interface enabling access results hits that can be visualized in (B) genome browser (JBrowse) with Liftoff tracks. Red arrows indicate region that are inconsistent between gene prediction and that might need curation and (C) in an orthogroup context with associated multiple alignments and phylogenetic tree

Users have multiple ways to search for genes in the system, either using a gene locus (or a list of them), keywords, genomic coordinates powered by MegaSearch [38] or using the BLAST graphical interface searches from Sequenceserver [39] (Figure 2A). Results are connected to genome browsers [37] specific to each genome. Comparisons between genomes are facilitated by tracks showing gene annotations projected on other genomes using the lift-over tool. It allows at a glance to see missing genes and investigate possible errors in the prediction of structural gene annotation [40] (Figure 2B).

Any gene search result lists several information including gene membership to orthogroups or gene families in *Musaceae*. The three versions corresponding to the *M. acuminata* reference genome (“DH Pahang” v1, v2 and v4) were conserved in the system for traceability. To enable orthogroup visualization, we developed extension modules that support visualisation of multiple genome alignment and phylogenetic tree with all functionalities provided by MSAviewer [41] and PhyloTree [42] respectively (Figure 2C).

**Figure 3:**
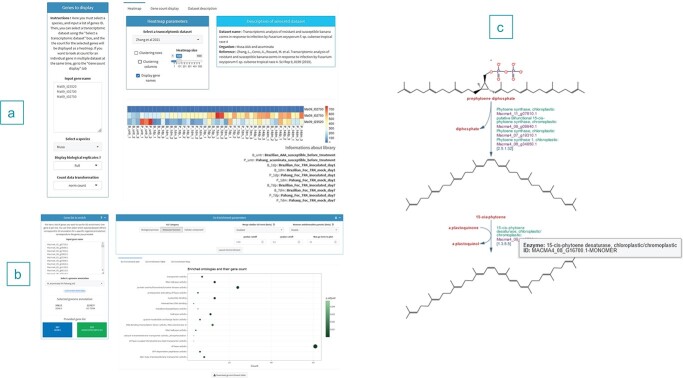
(A) Transcriptomic interface with a list of RGA2 genes from *M. acuminata* “DH Pahang” submitted to visualize their level of expression for a study on Fusarium wilt. (B) GO enrichment interface with a list of genes submitted. (C) First steps of the carotenoid pathways with Phytoene desaturase (PDS) identified by MusaCyc in the *Musa acuminata* genome.

For users interested in gene expression patterns for specific gene(s), we built interactive interfaces based on the shiny apps technology (R package) to enable manipulation of data results from published studies [29, 43, 44]. For instance, it is possible to search for genes annotated as RGA2, a putative nucleotide-binding and leucine-rich repeat (NB-LRR)-type resistance (R) gene known to be involved in the resistance to Fusarium wilt when overexpressed [45], and to check their level of expression in a study linked to Fusarium wilt [29] (Figure 3A).

Also, additional datasets can be uploaded in the Diane suite [46] to perform differential gene expression analyses, expression-based clustering and gene regulatory network analyses in which *Musa* references genomes were added. Besides, when a list of genes is identified, users can quickly test in a few clicks for Gene Ontology enrichment for several genomes and without the need to extract functional annotations and use external software (Figure 3B).

With regards to other OMICS, there have been increasing numbers of proteomics and metabolomics experiments in banana [30, 47–50]. To complement these resources and enable various options like experimental data overlay on metabolic pathways, we set up the latest version of PathwayTools v25 [51], named MusaCyc, that comprises a comprehensive set of interfaces to cover user needs. For instance, the carotenoid pathway has been actively studied in banana [52–54] and the Phytoene desaturase (PDS) enzyme, that can cause albinism when disrupted, was used as a proof of concept for gene editing. Using MusaCyc, the PDS gene can be easily found (Figure 3C).

### Genetic variant search and usage

**Figure 4:**
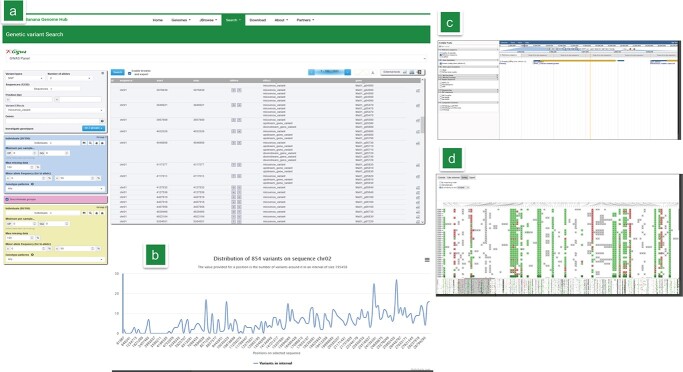
Overview of the genetic variant interface powered by GIGWA (A) Main interface for the GWAS panel with discriminated variants between 2 groups (seeded vs non-seeded) (B) Statistics of SNPs along Chromosome 2. (C) SNP visualization in JBrowse from the GIGWA interface (D) Data export online for graphical previews of genotype data in Flapjack-bytes.

This section, powered by the GIGWA tool [55, 56], gives access to a range of studies related to genetic diversity [57], GWAS [31, 33], Genomic selection or chromosome structure exploration [58, 59]. Notably, available studies include SNPs of the diploid banana panel that was designed specifically for GWAS analyses [31] while corresponding plant material for this panel can be ordered for phenotyping at the International Transit Center (ITC) via the *Musa* Germplasm Information System (MGIS) website [60, 61]. After filtering with advanced functionality, the datasets can be exported in multiple formats for subsequent analyses such as genetic diversity studies or directly visualized in JBrowse, IGV, Flapjack (and flapjack-bytes) (Figure 4). In addition, this catalogue of variants is compliant with BrAPI v1 & v2 [62] and can be accessed programmatically and used in third party client or databases.

### Pangenome viewer and exploration

**Figure 5:**
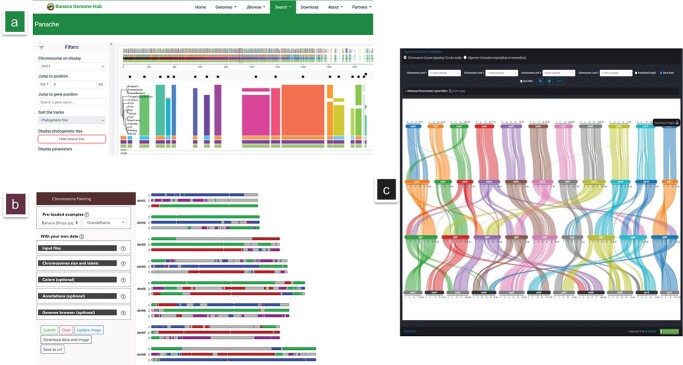
Overview of proposed web interfaces for comparative genomics within *Musaceae*. (A) Overview of the *Musaceae* Pangenome represented with the Panache interface. (B) Examples of genome ancestry mosaics. (C) Synteny between *Ensete glaucum*, *Musa acuminata*, *Musa balbisiana* and *M. schizocarpa* using Synvisio.

A single reference genome is not enough to capture genetic diversity in a species or a genus [63, 64]. To capture the diversity of gene content across *Musaceae*, a draft cross genus (*Musa-Ensete*) pangenome was built. It revealed distinct presence/absence patterns between genera [17]. While global results were analysed, exploration of specific regions along pan-chromosomes is still to be done. To make this easier, we implemented an instance of the Panache software [65] which enables the exploration of gene presence/absence variations (PAV) within pan-chromosomes. With it, users can automatically search for PAV areas and visualize them in the interface, where each line corresponds to one of the re-sequenced individuals (Figure 5A). Multiple sorting options (taxonomy, presence or absence of a given gene, etc.) are proposed to guide users toward genomic regions rich in PAV or showing a particular pattern.

### Genome ancestry mosaics viewer

Cultivated bananas result from a relatively limited number of sexual events with inter(sub) specific hybridizations and recombination [67]. The different ancestral contributions can be represented as genomic segments of distinct origin along the chromosomes. To provide access to recent studies that reported recombination between A and B genomes [59] and genome ancestry mosaics for a panel of diploid and triploid bananas [66], we embedded a new tool, called GeMo [67]. By selecting an samples like “Grande Naine” (AAA), an autotriploid cultivar belonging to the Cavendish subgroup, users can immediately spot the ancestral contributors of the *M. acuminata* subspecies, predominantly “banksii”, “zebrina”, “malaccensis” (Figure 5B). This viewer is intended to become a registry for any future studies performing in silico chromosome painting on Musaceae individuals but also enable user to manipulate their own data in a non-persistent way.

### Synteny viewer

The Zingiberales order evolution was shaped by lineage specific ancient whole genome duplications [7, 22] and within the *Musaceae*, for which the crown age was estimated at 59.19 Ma [68], a large number of chromosome rearrangements occurred [24, 69]. As an example, *M. acuminata* and *M. balbisiana* differ by a large translocation on chromosome1/3 and a large inversion on chromosome 5 [12]. To explore the chromosome structure between genome assemblies, SynVisio [70] was implemented for syntenic block visualization. It enables the comparison of two or more genomes (Figure 5C) and supports multi-resolution analysis and interactive filtering. Users can compare genomes one to one or in multi-genome mode. Conveniently, it also allows downloading high-quality images. Such a tool will be increasingly relevant as new assemblies are produced to visualize and understand fusion and fission events between chromosomes in *Musaceae* where different basic chromosome numbers exist (from 7 to 11 haploid chromosomes).

## Database construction and content

### Collection of genome assemblies and gene annotation

We collected 16 publicly released *Musaceae* nuclear genome sequences (8 high-quality and 8 draft sequences) that were released publicly (Table 1) as well as 91 chloroplast assemblies [68, 71–75]. Functional annotations from InterPro were obtained using InterProScan [76]. Gene ontology (GO) were retrieved by combining results from interpro2go and BlastP on SwissProt and TrEMBL [77]. For each assembly, they were compared and mapped using Liftoff [40]. When available, TE annotations from published studies were inserted into JBrowse.

Only minimal modifications of the assemblies or annotations from their description in publications are intended, to facilitate comparisons and traceability. In some cases, however, we improved the gene annotation: in agreement with data providers, we filtered *M. balbisiana* PKW for TE and released a new annotation; we also released a new annotation for *M. balbisiana* “DH PKW” where we reversed some chromosomes to be consistent with the orientation in *M. acuminata* “DH Pahang” and *Musa schizocarpa*.

### Transcriptomics and pathway related datasets

Transcriptomics data supplied by the community were included [12, 43, 44, 79, 81]. RNAseq data were mapped using STAR [82] and added in JBrowse as mapped tracks and in the download section. Whenever possible, derived reads count from published transcriptomics studies were collected and connected to the transcriptomics interface [29, 43, 44]. For pathway related information, enzymes and metabolic pathways were predicted from the protein-coding genes of *M. acuminata* “DH Pahang” v4. Enzyme Classification (EC) numbers were predicted combining both tools PRIAM [83] and BlastKOALA [84]. As a result, data were inferred for 774 pathways, 6762 enzymatic reactions and 97 transport reactions. A total of 8220 enzymes have been annotated and are available in the pathway tools section of the BGH.

### Comparative genomic analysis

We identified syntenic genes in the five chromosome scale assemblies available for *Musaceae*. Protein-coding genes were processed to identify reciprocal best hits (RBH) with BLASTP (e-value 1e-10) followed by MCScanX (e-value 1e-5, max gaps 25) [85].

### Gene family identification

Protein-coding genes from *E. glaucum* v1, *M. acuminata* (“DH Pahang” v2, Zebrina “Maia Oa”, “Calcutta 4” and “Banksii”), *M. balbisiana* v1.1 and *M. schizocarpa* v1 were processed using OrthoFinder v2.5.2 [86] with default parameters. We built the alignments and gene trees by applying our phylogenomic workflow, as implemented in GreenPhylDB [87].

### Genetic variants

SNP markers from multiple studies were retrieved and inserted into the GIGWA v2 genotyping database [55]. Quality checks, read mapping on reference genomes, SNP calling and variant effect in genic regions were conducted as described in [1]. The outputs of the analyses were produced in the variant call format (VCF), then loaded in GIGWA with associated metadata [55].

### Pangenome

Pangenome assembly, gene annotation and PAV matrix were collected from [17]. The study was based on 15 accessions across *Musa* and *Ensete* sequenced with short read technologies. To define the presence-absence of genes in the different accessions, they assembled the pangenome iteratively and annotated the genes in the new contigs, then proceeded with read mapping.

### Genome and transcriptome sequencing status

The curated list of SRA genomic resources was searched on NCBI SRA [88] by filtering on Taxonomic ids for *Musa* and *Ensete* and metadata was extracted from BioSample metadata descriptions. Information on ongoing projects was obtained by personal communications and interactions within the scientific community.

**Table 1:** List of genome sequence assemblies accessible via Banana Genome Hub. (CS: chromosome scale; SR: short reads; LR: long reads)

Species	Genotype	Version	Technology	Status	Comments	References
*Musa acuminata*	DH Pahang	1	Sanger + Illumina SR	High quality draft	1st reference (A genome)	[7]
*M. acuminata*	DH Pahang	2	Illumina SR + optical map	Improved high quality draft		[9]
*M. acuminata*	DH Pahang	4	Nanopore LR + Illumina	Telomere to telomere	Final version	[10]
*M. acuminata*	Banksii	2	Illumina + PacBio LR	Draft	CS in progress	[16]
*M. acuminata*	Maia Oa	1	Illumina SR	Draft	CS in progress	[16]
*M. acuminata*	Calcutta 4	1	Illumina SR	Draft	CS in progress	[16]
*Musa balbisiana*	PKW	1	Illumina SR	Draft		[11]
*M. balbisiana*	DH PKW	1.1	Illumina SR, PacBio LR + Hi-C	Chromosome scale	B genome reference	[12]
*Musa itinerans*	-	1	Illumina SR	Draft		[13]
*Musa schizocarpa*	-	1	Nanopore LR + Bionano	Chromosome scale	S genome	[18]
*Ensete glaucum*	-	1	Nanopore LR + Hi-C	Chromosome scale		[19]
*Ensete ventricosum*	Bedadeti	3	Illumina SR	Draft (download only)		[14, 78]
*Musa textilis (abaca)*	abuad	-	Illumina SR PacBio LR	Draft (download only)	CS in progress	[15]
*M. acuminata*	Dwarf Cavendish	1	Illumina SR	Draft (download only)		[79]
*Musa troglodytarum*	Karat	1	Nanopore LR + Illumina SR + PacBio LR + Hi-C	Chromosome scale		[80]
*Musa beccarii*		1	Nanopore LR + Hi-C	Chromosome scale		Early advance

## Discussion and perspectives

The Banana Genome Hub is a comprehensive platform dedicated to the genomics of a specific plant family – the *Musaceae* - as it has been developed for other families such as the Rosaceae [89] or the Juglandaceae [90]. The core functionalities are similar by providing access to genome datasets via JBrowse [91], BLAST, synteny and gene families viewers. However, the BGH has some specificities taking into account the nature of the plant and the existing ecosystems of tools and databases in the community.

An innovative pangenomics-related interface, Panache [65], has been implemented to support exploration of presence-absence variation (PAV). Both provides possible valuable resources for the design and exploration of precision genetics studies being conducted in the genus *Musa* [52, 92]. Besides, as a vegetatively propagated plant with low fertility, unravelling the genome ancestry mosaics of cultivated bananas has been initiated to decipher it complex domestication history [66] and we provide a unique way to store and visualize, through GeMo, future work in that direction. For functional oriented studies, users have now access to handy interface to check gene expression and functional enrichment.

Furthermore, the BGH intends to complement other databases on bananas and contribute to a better conservation and use of *Ensete* and *Musa* genetic resources. Contrary to the other portal [89, 90], the BGH does not intend to develop its own breeding module but rather proposes to implement BrAPI standards [62] to increase interoperability with the Banana instance of Breedbase [93]; which has been specifically designed for this purpose and that is actively supported by some banana breeding programs. Like GDR [89], a catalogue of variants is curated to provide facilitated access to data for SNP-based published studies. This catalogue, maintained by a different system, is shared with the Musa Germplasm Information System (MGIS) [60] to connect with the existing diversity of genetic resources conserved and documented in genebanks.

**Table 2:** Examples of genebanks or germplasm collection where material can be requested for research purposes

Collection name	Country	# Available Accessions	Distribution	Conditions	Access
International Transit Center (ITC)	Belgium	990	International	Free of charges (SMTA)	https://www.crop-diversity.org/mgis/moos/how-to-order
CRB Plantes Tropicales Antilles CIRAD-INRAe (CRB-PT)	Guadeloupe, France	381	International	Free except transport (SMTA)	http://crb-tropicaux.com/Portail
International Institute of Tropical Agriculture (IITA)	Nigeria	275	Regional (Africa)	Free of charges (SMTA)	https://www.genesys-pgr.org

While the *Musaceae* family contains 80 species classified in three genera, the Banana Genome Hub includes all publicly available whole genomes for eight species from two genera. Therefore, the BGH is designed to hold more whole genomes, and still has high potential to grow and to propose new tools to efficiently exploit new datasets considering specificities of the crop (e.g. polyploidy, structural variations). We will continue to curate and add new genome assemblies and related OMICS data as they become publicly available. Given the level of structural variation including chromosome rearrangements that are now well documented between the six species, high quality (N50 nearing average chromosome length) genome sequences (currently supported by Hi-C and/or long-molecule sequencing and genetic mapping data) are required as references.

To guide sampling for future sequencing projects and in an attempt to manage redundancy in data generation, we compile information from public sources or gleaned in conferences or from personal communications that will be regularly updated online (https://banana-genome-hub.southgreen.fr/content/sequencing-status). The first observation is that if no genome assembly of known *Musa* cultivars, mostly triploids, has been released at chromosome-scale, some are underway as well as for additional wild species. Increasing accuracy of long-molecule sequencing is important to assembling haplotypes in triploid hybrids that are so important regionally and in trade. High quality whole-genome assemblies underpin exploitation of survey sequence data for allele mining or GWAS (Genome Wide Association Studies) to identify functional variants. Re-sequencing is ongoing in several germplasm collections, which will help identifying allelic and potentially copy number variation. Also, assemblies are available for chloroplast genomes on wild species, sometimes redundantly, and future effort might focus on cultivated groups and systematically cover the diversity of the family.

Whenever possible, plant material used to generate genomic data should be deposited in genebanks or national collections (Table 2) where passport data, possibly associated with phenotype information, is documented and material distribution processes are streamlined. For instance, use of accessions from the International Transit Center (ITC) [60, 61] or the CRB Plantes Tropicales Antilles CIRAD-INRAe can facilitate traceability, reproducibility, and data integration with previous and future experiments since accessions can be sent internationally, virus indexed and free of charge for research purposes. Furthermore, missing accessions of interest can be also proposed to ITC for conservation.

Regarding gene annotation, we recommend adopting a defined nomenclature for locus tag that would consider the wide range of wild *Musaceae* species (Table S1). However, we acknowledge that further work is necessary to address the case of groups and subgroups in cultivated bananas.

Finally, we encourage scientists generating genomics data in Musaceae to contact us or the Genomics Thematic group of MusaNet (https://musanet.org) early in the publication process to make sure that general standards (chromosome orientation, gene locus) are consistent with existing resources and eventually to get support to create dedicated pages and associated tools (BLAST, JBrowse, download).

## Acknowledgements

This work was partially supported of the CGIAR Research Program on Roots, Tubers and Bananas (RTB), the Agropolis Foundation (ID 1504-006) “GenomeHarvest” project through the French Investissements d’avenir programme (Labex Agro: ANR-10-LABX-0001-01). XJ. G. acknowledges support of the National Natural Science Foundation of China (No. 32070237, 31261140366). This work is technically supported by the South Green Bioinformatics platform and the CIRAD - UMR AGAP HPC Data Center. We warmly thank all data providers who proactively enrich the BGH with datasets and feedback including Sebastien Carpentier, Julie Sardos, Sijun Zheng, Nicolas Roux (Alliance Bioversity International - CIAT), David Studholme (University of Exeter), Boas Pucker (CeBiTec), Chunyan Xu, Xiaodong Fang (BGI), Ana Almeida (California State University East Bay), Wei Hu (CATAS), Mark Davey (KU Leuven), Dave Edwards, Philipp Bayer (University of Western Australia), Jose de Vega (Earlham Institute). We are grateful to Gabriel Sachter-Smith, Pat Heslop-Harrison, Julie Sardos, Ziwei Wang and Megan Hansen who provided the beautiful pictures for the homepage.

## Author Contributions

M.R. and G.D. designed and managed the project. G.D. constructed the core database; V.G., M.S., E.D., G.S. developed additional modules. G.D., G.M., F-C.B., C.B. and M.R. collected and analysed datasets. P. H-H., T.S., XJ. G., N.Y., A.DH. supported the Hub with key resources. M.R. drafted the manuscript, and all authors were involved in manuscript revision and approved the submitted version.

## Data availability statement

For data download, the BGH is structured by organism with regards to individual genome assemblies and also by studies that provide directory listing of the related datasets. A global download section, supported by Drupal Filebrowser module, provides FTP-like browsing capabilities for datasets (e.g. FASTA, GFF, BAM/CRAM, VCF). The catalogue of variants can also be accessed using Breeding API (BrAPI) [62]. The BGH is proposed as a FAIR (Findable, Accessible, Interoperable and Re-usable) compliant resource [94] (https://bio.tools/Banana_Genome_Hub), and according to FAIR checker (https://fair-checker.france-bioinformatique.fr/check), it scored a high level in terms of accessibility and findability (Figure S1).

## Conflict of interests

The authors declare that they have no conflict of interest.

## Supplementary data

Supplementary data is available at *Horticulture Research* online.

## Supplementary Material

Web_Material_uhac219
