# Supplementary material for: The banana genome hub: a community database for genomics in the Musaceae
Source: Hortic Res. 2022 Sep 28;9:uhac221. doi: 10.1093/hr/uhac221 (PMC9720444; doi:10.1093/hr/uhac221)
Supplement: Web_Material_uhac219 [file web_material_uhac219.zip › Supplementary.docx]

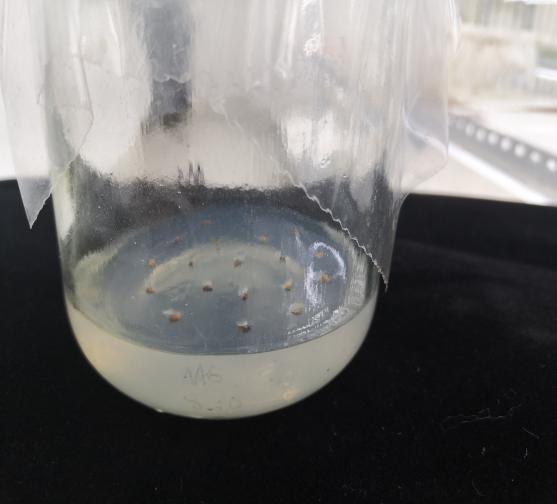

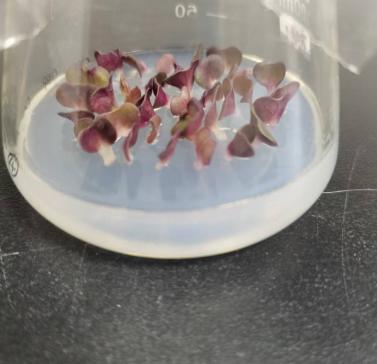

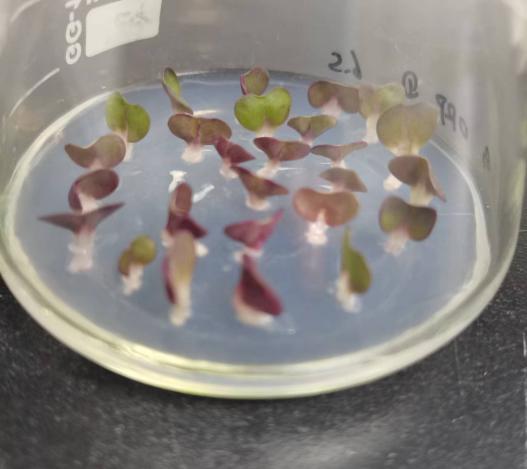

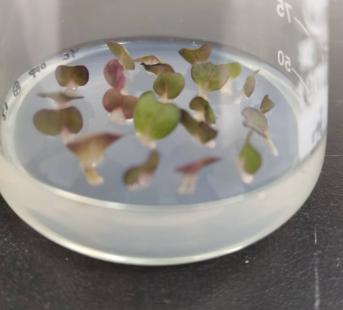

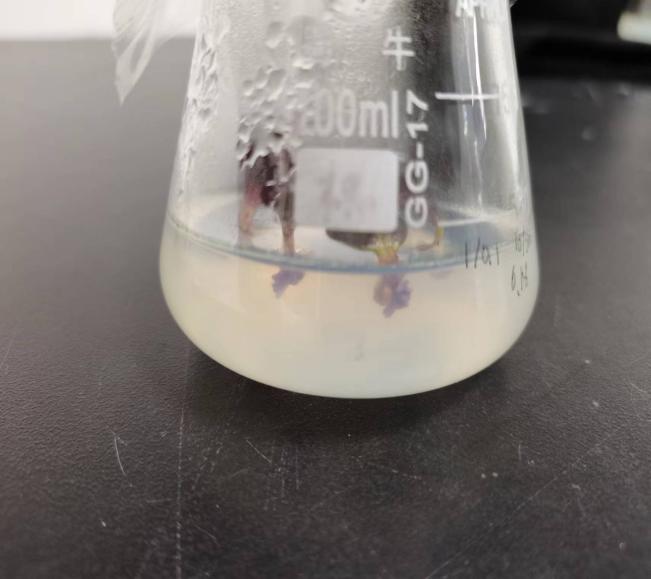

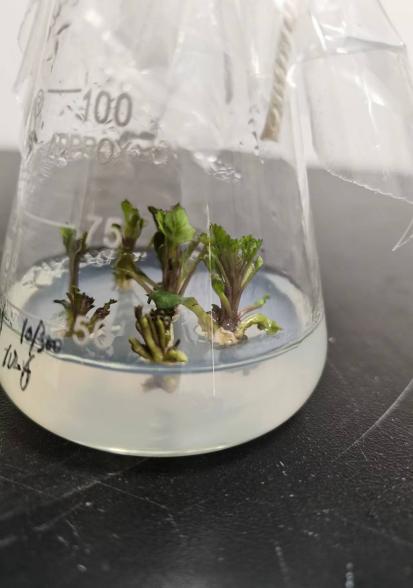


**c**

**b**

**a**

**f**

**e**

**d**

Figure S1 The process of *Agrobacterium*-mediated transformation in plants. a Sowing period. b Pre-cultivation. c Co-cultivation. d Delayed culture. e Screening culture. f Rooting culture.

Figure S2 Comparison of the amino acid sequences of *Bo1g144580*, *Bo3g062100*, *Bo5g139390* and *BoORP3a* (*Bo9g184810*).

Figure S3 Multiple alignment of ORP3a [amino](F:/Youdao/Dict/7.5.2.0/resultui/dict/?keyword=amino) [acid](F:/Youdao/Dict/7.5.2.0/resultui/dict/?keyword=acid) [sequence](F:/Youdao/Dict/7.5.2.0/resultui/dict/?keyword=sequence) from 10 species of Cruciferous plants.

Figure S4 Comparison of the genomic sequence of *BoORP3a* (*Bo9g184810*) between the S0835 and the F0819.

Figure S5 Comparison of the coding sequence of *BoORP3a* (*Bo9g184810*) between the S0835 and the F0819.

Figure S6 Comparison of the amino acid sequence of *BoORP3a* (*Bo9g184810*) between the S0835 (WT) and [mutant](F:/Youdao/Dict/7.5.2.0/resultui/dict/?keyword=mutant) plants (*orp3a-1*, *orp3a-21* and *orp3a-34*).

Table S1 Primer sequence for clone, subcellular localization, vector construction and transgenic plants test.

|  | Genes | Primer sequence (5’--3’) | Amplicon length (bp) |
| --- | --- | --- | --- |
| Clone  primers | *BoORP3a* | F-ATGTCTCCTAACGATTCAAAAAAC | 2180 |
|  |  | R-TTAAGGAGAGGTATCTTGGAAC |  |
| Subcellular localization  primers | *BoORP3a* | F-gaacacgggggactcttgacATGTCTCCTAACGATTCAAAAAAC |  |
|  |  | R-cctttactagtcagatctacAGGAGAGGTATCTTGGAAC |  |
| sgRNA  primers | ORPDT1-BsF | ATATATGGTCTCGATTGACCGACCCGAAGTTGGTGAGTT |  |
|  | ORPDT1-F0 | TGACCGACCCGAAGTTGGTGAGTTTTAGAGCTAGAAATAGC |  |
|  | ORPDT2-R0 | AACGCCAATGAGTGCTGCTCACCAATCTCTTAGTCGACTCTAC |  |
|  | ORPDT2-BsR | ATTATTGGTCTCGAAACGCCAATGAGTGCTGCTCACCAA |  |
| CRISPR test  primers | ORP-test1-F1 | ACCGTCAAGGAAGACCAG | 440 |
|  | ORP-test1-R1 | TCTTCCTGCTTCCTCCTC |  |
|  | ORP-test2-F2 | ATCCAATCCTCGGTGAAAC | 394 |
|  | ORP-test2-R2 | CAAGAACCACACCATCTCT |  |
